# Complete Closed Genome Sequence of Nontoxigenic Invasive *Corynebacterium diphtheriae* bv. mitis Strain ISS 3319

**DOI:** 10.1128/genomeA.01566-17

**Published:** 2018-02-01

**Authors:** Camila Azevedo Antunes, Emily J. Richardson, Joshua Quick, Pablo Fuentes-Utrilla, Georgia L. Isom, Emily C. Goodall, Jens Möller, Paul A. Hoskisson, Ana Luiza Mattos-Guaraldi, Adam F. Cunningham, Nicholas J. Loman, Vartul Sangal, Andreas Burkovski, Ian R. Henderson

**Affiliations:** a Microbiology Division, Friedrich-Alexander University Erlangen, Nuremberg, Erlangen, Germany; b Laboratory of Diphtheria and Corynebacteria of Clinical Relevance–LDCIC, Faculty of Medical Sciences, Rio de Janeiro State University, Rio de Janeiro, Brazil; c Institute of Microbiology and Infection, School of Biosciences, University of Birmingham, Birmingham, United Kingdom; d Strathclyde Institute of Pharmacy and Biomedical Science, University of Strathclyde, Glasgow, United Kingdom; e Faculty of Health and Life Sciences, Northumbria University, Newcastle upon Tyne, United Kingdom

## Abstract

The genome sequence of the human pathogen *Corynebacterium diphtheriae* bv. mitis strain ISS 3319 was determined and closed in this study. The genome is estimated to have 2,404,936 bp encoding 2,257 proteins. This strain also possesses a plasmid of 1,960 bp.

## GENOME ANNOUNCEMENT

*Corynebacterium diphtheriae* is the etiological agent of diphtheria, a toxigenic infection of the upper respiratory tract. Besides diphtheria, systemic infections caused by nontoxigenic strains have been reported (1–5). The molecular basis of toxin-independent pathogenicity of *C. diphtheriae* is poorly understood (6). Therefore, we sequenced the whole genome of *C. diphtheriae* bv. mitis ISS 3319, isolated from the throat of 9-year-old patient (7, 8). The strain lacks the *tox* gene but is able to adhere to and invade human epithelial cells and effectively colonize spleen and kidneys of mice (9–11).

Sequencing of libraries was carried out with the Oxford Nanopore MinION instrument and the Illumina HiSeq platform using a 250-bp paired-end protocol. The Illumina and MinION reads were assembled together using Unicycler with Pilon polishing, with a final hybrid assembly of two contigs of 1,604,547 and 800,125 bp. Two gaps of 124 bp and 140 bp were identified in the genome using BLAST searches of >500 bp from the ends of both the contigs against the published assembly of the same strain (GenBank accession no. JAQN00000000). The contigs were merged using the missing sequence after aligning them to the genome sequence of strain C7 (beta) (GenBank accession no. NC_016801), which belongs to the same sequence type, ST26, as ISS 3319 (7). Automatic annotation and identification of rRNAs and tRNAs were accomplished using the Prokka version 1.11 software tool (12).

The new sequence was compared to the previously published assembly of ISS 3319, which was also reannoated using Prokka to obtain an equivalence of annotation for comparative analysis. The annotated genomes were compared using Roary (13, 14); 2,195 genes are conserved between the assemblies, and only four single nucleotide polymorphisms (SNPs) were observed in the core genomic alignment. Most of the 26 genes reported to be specific to the previous assembly (GenBank accession no. JAQN00000000) showed significant similarities to several genes in the new assembly and may not be specific. The proteins encoded by the majority of these genes are short and fall within or near the identified genomic islands that were identified using IslandViewer 4 (15).

The closed genome of 2,404,936 bp contained 9 rRNAs, 53 tRNAs, 1 transfer-messenger RNA, 1 repeat region, 2,257 protein-coding sequences, and 53.46% GC content. Moreover, a circular plasmid of 1,960 bp with four coding sequences was also identified. A discontinuous mega-BLAST search of this plasmid sequence in GenBank revealed partial similarities (67% identity in 612-bp alignment) with plasmid pTA144 in *Moraxella* sp. (GenBank accession no. NC_001316) and the metamobilome of an uncultured strain (65% identity in 833-bp alignment; GenBank accession no. LN853682). A nonredundant translation alignment of the plasmid sequence showed a conserved domain with homology to the phage replication protein Cri, described as essential for DNA replication in *Vibrio cholerae* serotype O1 (16), and a sequence with 88% identity to a gene encoding an αβ-hydrolase from a *Streptococcus* sp. (GenBank accession no. WP_002883881). Coinfections of *C. diphtheriae* with other pathogens, such as *Staphylococcus* spp., *Streptococcus* spp., *Arcanobacterium* spp., and *Pseudomonas* spp., were reported previously (8, 17, 18), and this close contact might support horizontal DNA transfer.

### Accession number(s).

The *C. diphtheriae* bv. mitis ISS 3319 whole-genome shotgun project has been deposited at DDBJ/EMBL/GenBank under the accession no. CP025209 and CP025210. The versions described in this paper are the second versions.
